# tRNA-derived fragments: Mechanisms underlying their regulation of gene expression and potential applications as therapeutic targets in cancers and virus infections

**DOI:** 10.7150/thno.51963

**Published:** 2021-01-01

**Authors:** Xiuchong Yu, Yaoyao Xie, Shuangshuang Zhang, Xuemei Song, Bingxiu Xiao, Zhilong Yan

**Affiliations:** 1 Department of Biochemistry and Molecular Biology and Zhejiang Key Laboratory of Pathophysiology, Medical School of Ningbo University, Ningbo 315211, China.; 2 Department of Gastrointestinal Surgery, Ningbo First Hospital, Ningbo University, Ningbo 315010, China.

**Keywords:** tRNA-derived fragment (tRF), mechanism, cancer, virus infection, therapeutic strategy

## Abstract

tRNA-derived fragments (tRFs) are a new category of regulatory noncoding RNAs with distinct biological functions in cancers and stress-induced diseases. Herein, we first summarize the classification and biogenesis of tRFs. tRFs are produced from pre-tRNAs or mature tRNAs. Based on the incision loci, tRFs are classified into several types: tRF-1, tRF-2, tRF-3, tRF-5, and i-tRF. Some tRFs participate in posttranscriptional regulation through microRNA-like actions or by displacing RNA binding proteins and regulating protein translation by promoting ribosome biogenesis or interfering with translation initiation. Other tRFs prevent cell apoptosis by binding to cytochrome* c* or promoting virus replication. More importantly, the dysregulation of tRFs has important clinical implications. They are potential diagnostic and prognostic biomarkers of gastric cancer, liver cancer, breast cancer, prostate cancer, and chronic lymphocytic leukemia. tRFs may become new therapeutic targets for the treatment of diseases such as hepatocellular carcinoma and respiratory syncytial virus infection. Finally, we point out the existing problems and future research directions associated with tRFs. In conclusion, the current progress in the research of tRFs reveals that they have important clinical implications and may constitute novel molecular therapeutic targets for modulating pathological processes.

## Introduction

**Figure 1:**
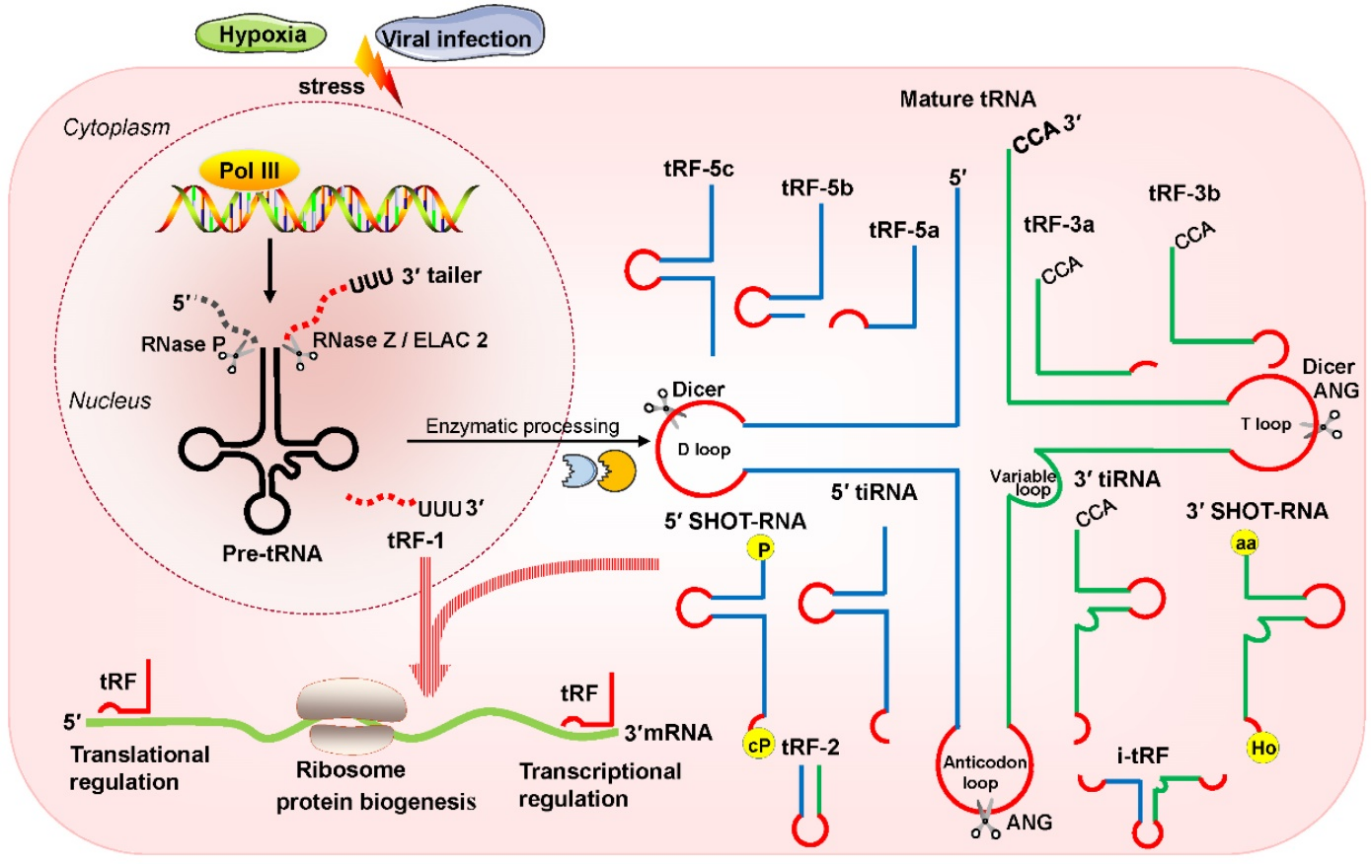
Classification and biogenesis of tsRNAs. tRFs can be classified into five types: tRF-5, tRF-3, tRF-1, tRF-2, and i-tRF. tiRNAs are cleaved by ANG at the middle site of the anticodon of mature tRNAs. SHOT-RNAs are induced by sex hormones. The production of tRFs and tiRNAs is promoted by various stress conditions. They play roles in processes such as transcriptional regulation and the regulation of protein biogenesis. Abbreviations: tsRNAs, tRNA-derived small RNAs; Pol III, polymerase III; pre-tRNA, precursor tRNA; RNase Z, ribonuclease Z; ELAC2, cytoplasmic homolog ribonuclease Z 2; ANG, Angiogenin; SHOT-RNAs, sex hormone-dependent tRNA-derived RNAs; tRF, tRNA-derived fragments; tiRNA, tRNA-derived stress-induced RNA; P, phosphate; cP, cyclicphosphate; aa, amino acid; Ho, hydroxyl.

Recently, multiple studies have revealed that small noncoding RNAs (sncRNAs) exist extensively and have a diversity of functions in humans 1-3. In the eukaryotic nucleus, RNA polymerase III (RNA Pol III) plays the role of transcribing tRNA genes into precursor tRNAs (pre-tRNAs) 4, 5. During tRNA maturation, the typical cloverleaf structure of the pre-tRNA 5'-leader sequence as well as the 3'-tail ('poly-U') sequence are enzymatically digested by endoribonuclease P (RNase P) and endonuclease Z (RNase Z)/cytoplasmic homolog ribonuclease Z 2 (ELAC2), respectively; then, under the action of tRNA nucleotidyl transferase, the trinucleotide 'CCA' sequence is attached to the tail-free tRNAs at the 3' ends 4, 5. The tRNA transcripts undergo enzymatic splicing, and chemical modification may yield new species of sncRNAs, such as tRNA-derived small RNAs (tsRNAs) 6, 7. According to the cleavage loci and length, tsRNAs can be divided into two major categories: tRNA-derived stress-induced RNAs (tiRNAs) and tRNA-derived fragments (tRFs) (**Figure 1**). The generation of tiRNAs and tRFs takes place in multiple biological processes, which implies that these fragments are not random cleavage products 7.

tiRNAs are generated by ribonuclease angiogenin (ANG) incision at the middle site of the anticodons of mature tRNAs, so tiRNAs are also called tRNA halves 8, 9. tiRNAs, with lengths of 31- 40 nucleotides (nt), have the 5′ hydroxyl but not the 5′ phosphate, which is different from the situation for microRNA (miRNA) 5′ phosphate ends, which are generated by Dicer enzymes 8, 9. Based on whether the 5′ or 3′ sequencer of the anticodon cleavage position is included, tiRNAs are classified into two basic types: 5′ tiRNAs and 3′ tiRNAs 8, 9. The 5′ tiRNAs encompass the 5′ end of the mature tRNA to the terminus of the anticodon loop, while the 3′ tiRNAs extend from the anticodon loop to the 3′ end of the mature tRNA (**Figure 1**). These tiRNAs are mainly generated by stress exposure, such as oxidative stress, hypoxia, and virus infection 8, 9.

In addition, another type of tRNA half known as the sex hormone-dependent tRNA-derived RNA (SHOT-RNA) is induced by sex hormones and cleaved by ANG 10, 11. SHOT-RNA, which is not induced by stress, is particularly highly expressed in androgen receptor (AR)-positive prostate cancer cells and estrogen receptor (ER)-positive breast cancer and is not expressed in other cancers at present. Specifically, 5′ SHOT-RNAs bear a phosphate at the 5′ end and a 2′,3′-cyclicphosphate at the 3′-end (**Figure 1**), while 3′ SHOT-RNAs are characterized by a hydroxyl at the 5′ end and an amino acid at the 3′ end, as they are derived from aminoacylated tRNAs 10, 12, 13.

High-throughput sequencing technology has revealed that there is more diversity in tsRNAs than is reflected in the existing classifications. Among tsRNAs, tRFs, with a length of 14 nt to 30 nt, have a greater number of distinct classes and function as key players in the regulation of gene expression at transcriptional as well as posttranscriptional levels, indicating that they are not merely byproducts of the random cleavage of tRNAs but are regulatory sncRNAs involved in physiological and pathological processes 1, 3.

Since tRFs are the major types of tsRNAs, in this review, we focused on tRFs. We present updated views focusing on the specific molecular mechanisms underlying tRF-mediated regulation of mRNA stability and translation, stress responses and viral infections and their potential roles as biomarkers or therapeutic targets in cancers and virus infections.

## Classification and biogenesis of tRFs

tRFs are produced from pre-tRNAs or mature tRNAs 14, 15. Notably, they are similar in size to miRNAs, with a 5′ phosphate and a 3′ hydroxyl 14. According to the mapped locations, tRFs are largely categorized into two main classes: tRF-5 and tRF-3 14, 15. tRF-5s begin at the 5′ end of mature tRNAs and are cleaved by Dicer at the D-loop or the stem position between the D-loop and the anticodon loop 14, 15. On the basis of the incision loci and lengths, tRF-5s are further classified into three subtypes: tRF-5a (14-16 nt), tRF-5b (22-24 nt) and tRF-5c (28-30 nt) 14, 15. tRF-3s begin at the 3′ end (at the trinucleotide 'CCA' at the 3′ end) and are cleaved by Dicer and ANG at the T-loop of mature tRNAs. tRF-3s, which are approximately 18-22 nt in length, are further classified into two subgroups: tRF-3a and tRF-3b (**Figure 1**).

There are three additional groups of tRFs: tRF-1, tRF-2, and inter tRF (i-tRF) 15, 16. tRF-1s are small fragments derived from the 3′ tails of pre-tRNsA (containing the 'poly-U' sequence at the 3′ ends) and are cleaved by RNase Z or ELAC2 15. tRF-2s comprise anticodon loop and stem sequences, excluding the 5′ end and 3′ end structures 15, 16. However, the details of ribonuclease processing required for the production of tRF-2s and i-tRFs remain unclear 15, 16. i-tRFs, originating from the internal body of mature tRNAs 17, 18, include the anticodon loop and segments of the D-loop and T-loop other than the 5′ terminal and 3′ terminal (**Figure 1**).

## Regulation of mRNA stability

Studies have revealed that some tRFs target genic mRNAs and participate in posttranscriptional regulation in humans 19. Here, we elaborate on tRFs targeting mRNA expression through miRNA-like actions and binding to RNA-binding proteins (RBPs) to control mRNA stability.

### MiRNA-like actions

Preparatory bioinformatic analysis suggests that tRFs have sufficient sequence complementarity with endogenous mRNAs and thus may play a potential role in posttranscriptional regulation 19, 20. As one kind of sncRNA with a length less than 30 nt, it seems logical to conclude that tRFs have functions similar to those of miRNAs 21, 22.

**Figure 2:**
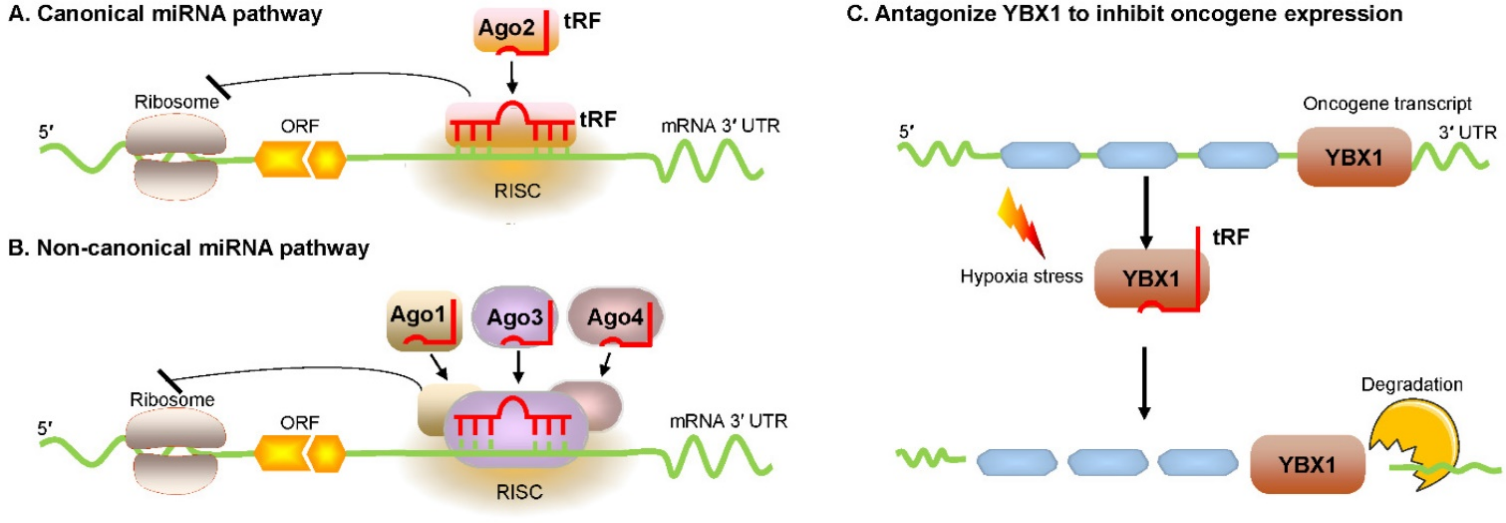
Regulation of mRNA stability by tRFs. (A, B) tRFs bind with different Ago proteins to inhibit targeted mRNA expression via pathways that exert either canonical or noncanonical miRNA-like activity. (C) tRFs cause oncogene transcript degradation by displacing YBX1 from the 3′ UTR of mRNA. Abbreviations: miRNA, microRNA; Ago, Argonaute; YBX1, Y-box-binding protein 1; 3′ UTR, 3′ untranslated region; RISC, RNA-induced silencing complex; ORF, open reading frame.

Huang et al. reported that tRF/miR-1280, which is derived from tRNA^Leu^ and pre-miRNA, inhibited colorectal cancer cell proliferation by inhibiting the Notch signaling pathway by directly interacting with the JAG2 mRNA 3′ untranslated region (UTR) 23. In particular, these tRFs are physically related to Argonaute (Ago) proteins, which regulate gene expression 24, 25. Recently, an increasing number of researchers have found that tRFs are loaded into Ago complexes 26, 27. RNA-seq analysis proves that tRF-3009a guides Ago to inhibit the expression of targeted genes in a Dicer-independent manner posttranscriptionally 28. Maute et al. verified in B cell lymphoma that tRF-3 derived from tRNA^Gly-GCC^ possessed an miRNA-like structure and functions by binding a complementary target site 29. Using large-scale meta-analyses of available experimental data, researchers observed Ago1-loaded tRFs, and these tRFs interacted with target genes at the 3ʹ UTR 30. Li et al. found that tRF-3s could guide Ago2 to cleave the target mRNA (**Figure 2A**) 31. Kumar et al. intriguingly reported that tRF-5s and tRF-3s were preferentially associated with Ago1, Ago3, and Ago4 but not with Ago2 in human embryonic kidney 293 (HEK293) cells (**Figure 2B**) 32. Hence, in conjunction with different Ago proteins, tRFs can regulate gene expression through either canonical or noncanonical miRNA-like actions 32-35. Additionally, Ago-bound tRFs should be further explored because they have a propensity to target endogenous mRNA and to construct regulatory networks in humans 36, 37.

### Bind to RNA-binding proteins

tRFs may bind to RBPs and posttranscriptionally regulate gene expression 38, 39. RBPs interact with targeted RNAs to control their stability 40.

In breast cancer cells, Goodarzi et al. reported that under hypoxic conditions, several tRFs were upregulated, which then suppressed oncogenic transcript stability by displacing the 3' UTR from the YBX1 protein 41. These hypoxic stress-induced tRFs, which are mainly derived from tRNA^Asp^, tRNA^Glu^, tRNA^Gly^, and tRNA^Tyr^, can competitively bind to YBX1 and block its interaction with oncogenic mRNAs 41. YBX1 is an RBP with many biological roles. YBX1 maintains oncogene transcript stability and increases cell proliferation by binding with endogenous oncogenic mRNA 42. YBX1 may also bind with several categories of regulatory RNAs, including tRFs 41. Under hypoxic stress, cancer cells could produce more tRFs that can compete with oncogene transcripts and then bind to YBX1, thereby promoting mRNA degradation and eventually inhibiting the proliferation of cancer cells (**Figure 2C**). This posttranscriptional suppression depends on sequence complementary because tRFs have a motif that binds the sequence that YBX1 can recognize 41, 43.

In fact, YBX1 is not the only RBP that may be displaced by specific tRFs. In fact, a novel tRF derived from mature tRNA^Glu^ has been found to be able to bind and displace the RBP nucleolin in breast cancer 44.

## Regulation of protein translation

tRFs play additional biological roles by activating or inhibiting protein synthesis via different mechanisms 18.

### Translational activation via promotion of ribosome biogenesis

**Figure 3:**
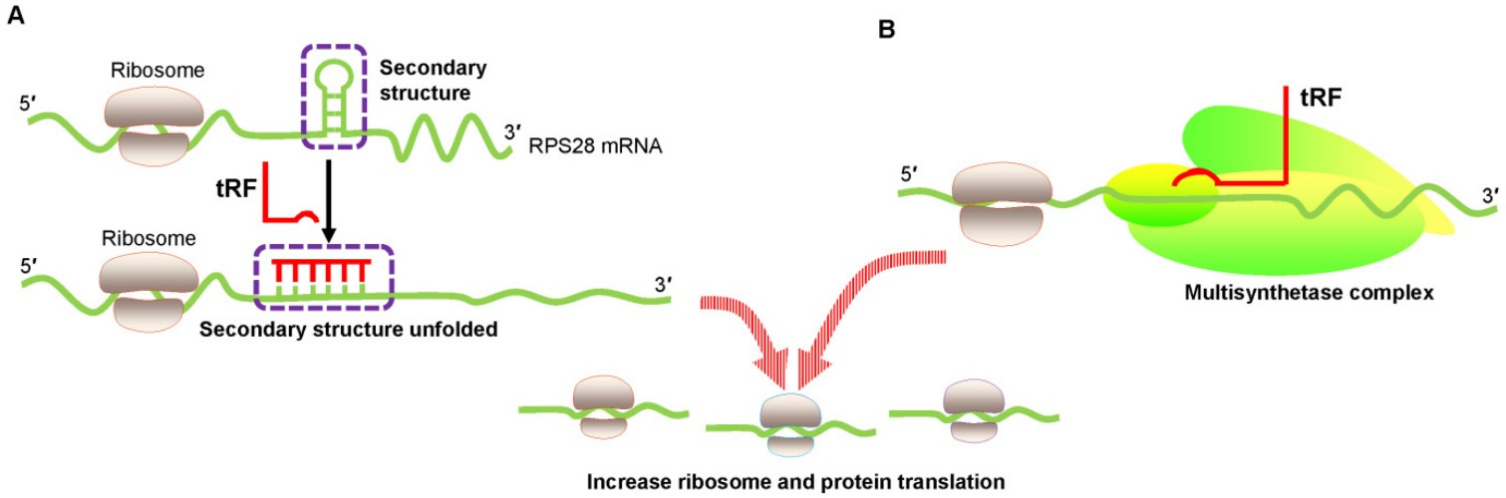
Translational activation by promoting ribosome biogenesis. (A) tRF increases ribosome biogenesis by unfolding the RPS28 mRNA secondary structure, thus enhancing translation. (B) tRF interacts with the human multisynthetase complex, thereby promoting RNA-binding protein translation. Abbreviations: RPS28, ribosomal protein S28.

An innovative study by Kim et al. proved that a 22 nt length tRF called 3′ tRF^LeuCAG^ enhanced translation by facilitating ribosome protein biogenesis 45. Ribosome gradient analysis showed that ribosomal protein S28 (RPS28) was needed for ribosomal RNA 18S rRNA biogenesis and was an integral part of the 40S ribosomal subunit 46. The 3′ UTR target site of RPS28 mRNA forms a secondary structure that is a major region containing a translation initiation site. Several experiments involving target-site mutations demonstrated that 3′ tRF^LeuCAG^ bound to duplexed secondary target sites in RPS28 mRNA and unwound the hairpin secondary structure to increase translation in human cancer cells 45 (**Figure 3A**). Thus, 3′ tRF^LeuCAG^ plays a vital function in regulating the numbers of ribosomes. The greater the number of ribosomes, the more potential protein synthesis, eventually increasing cell growth and proliferation 45.

Keam et al. also convincingly showed the translational activation of tRFs 47. 5′ tRF^Gln19^ interacted with the human multisynthetase complex (MSC) and then promoted RBP translation (**Figure 3B**). However, the detailed mechanism needs to be determined.

### Translational inhibition by interfering with translation initiation

**Figure 4:**
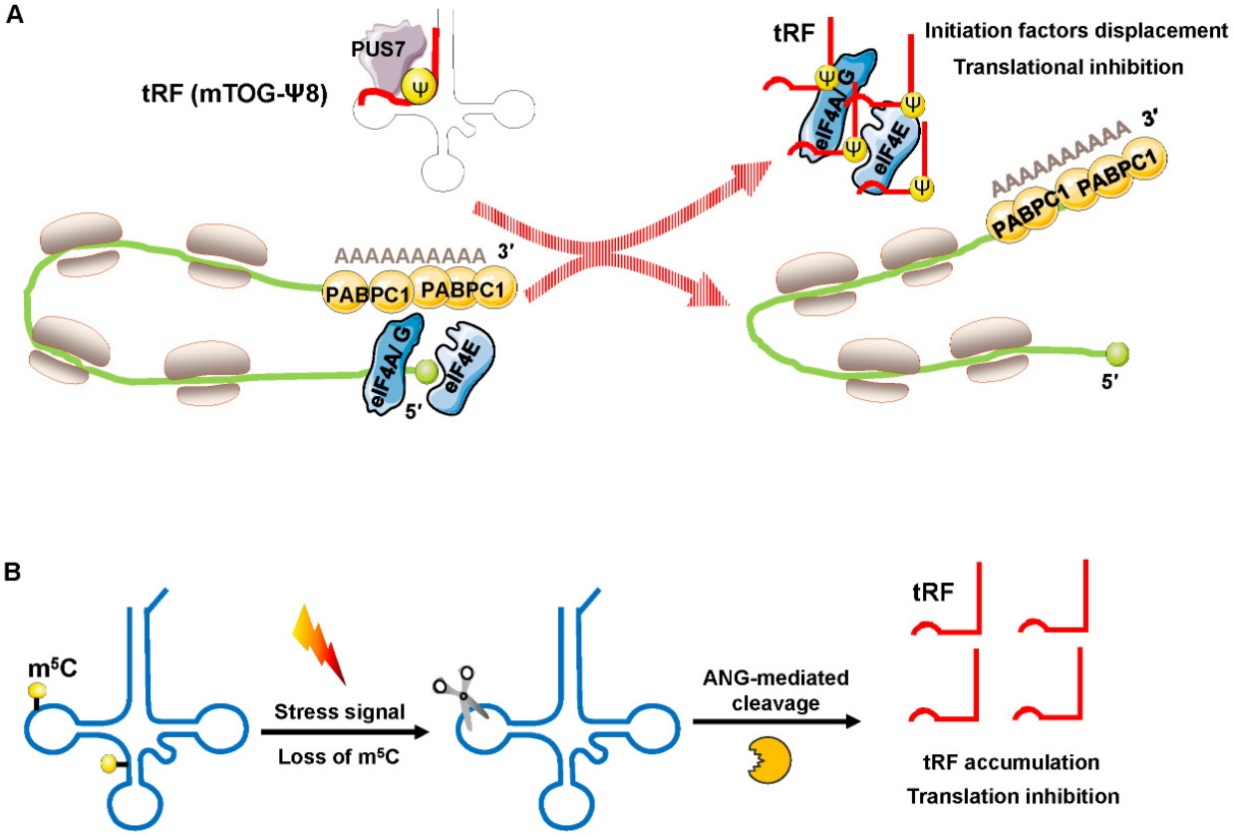
Translation inhibition by interfering with translation initiation. (A) Increased displacement of translational initiation factors from mRNA by the tRF mTOG-Ψ8 represses translation. Loss of PUS7 and mTOG-Ψ8 may contribute to human myeloid malignancies. (B) Loss of m^5^C increases the affinity between tRNA and ANG, leading to tRF-5 accumulation, which then decreases protein synthesis. Abbreviations: PUS7, pseudouridylation synthase 7; Ψ, pseudouridine; eIF4A/G, E, eukaryotic initiation factor 4 A/G, E; PABPC1, poly(A) binding protein-1; ANG, angiogenin.

Recently, Guzzi et al. showed that in stem cells, pseudouridylation synthase 7 (PUS7) mediated pseudouridine (Ψ) disposition of particular tRFs (mTOG-Ψ8) to suppress translation (**Figure 4A**). They identified that PUS7 was enriched in embryonic and/or hematopoietic stem cells and then bound to diacritical tRNAs and modified uridine (U) into pseudouridine (Ψ) at the U8 position (Ψ8) 48. PUS7-mediated “Ψ” controls the stem cell biogenesis of 5′ tRFs, which have a common oligoguanine motif at the terminus and are named mTOG-Ψ8 48. Under normal growth conditions, polysomes form a closed-loop translation complex (**Figure 4A**). In this structural model, translational initiation factors [eukaryotic initiation factor (eIF)-4 A/G, and E] interact with cytoplasmic poly(A) binding protein-1 (PABPC1) 49, 50. In response to stress, PUS7 binds to diacritical tRNAs and governs biogenesis of mTOG-Ψ8; then, mTOG-Ψ8 preferentially binds to PABPC1, resulting in displacement of eIF-4 A/G and E from m^7^G-capped mRNAs (**Figure 4A**). PUS7 and mTOG-Ψ8 loss affects translation regulation, resulting in the increased biosynthesis of proteins and the impairment of hematopoietic stem cell commitment, potentially leading to myeloid malignancies 48.

Blanco et al. demonstrated that posttranscriptional methylation of tRNA at cytosine-5 (m^5^C) by the methyltransferase NSUN2 was an innovative mechanism to suppress global protein synthesis 51. External stress stimulation represses NSUN2 activity, causing the loss of m^5^C; the dysregulation of m^5^C increases the affinity between tRNA and angiogenin, leading to the accumulation of tRF-5s, which then repress protein synthesis and promote squamous tumorigenesis (Figure 4B). Sobala et al. showed that tRF-5 derived from tRNA^Gln^ was able to inhibit translation and did not require complementary target sequences of mRNA 52. Mechanistically, these inhibitory effects of tRFs on translation require a conserved 3′ “GG” dinucleotide 52.

## Signs of cell stress and promotion of virus replication

The production of tRFs can be induced by stress conditions, such as high salinity, oxidative stress, and virus infection 53, 54. Persistently activated stress responses result in inflammation and disease pathogenesis 54.

### Signs of cell stress

**Figure 5:**
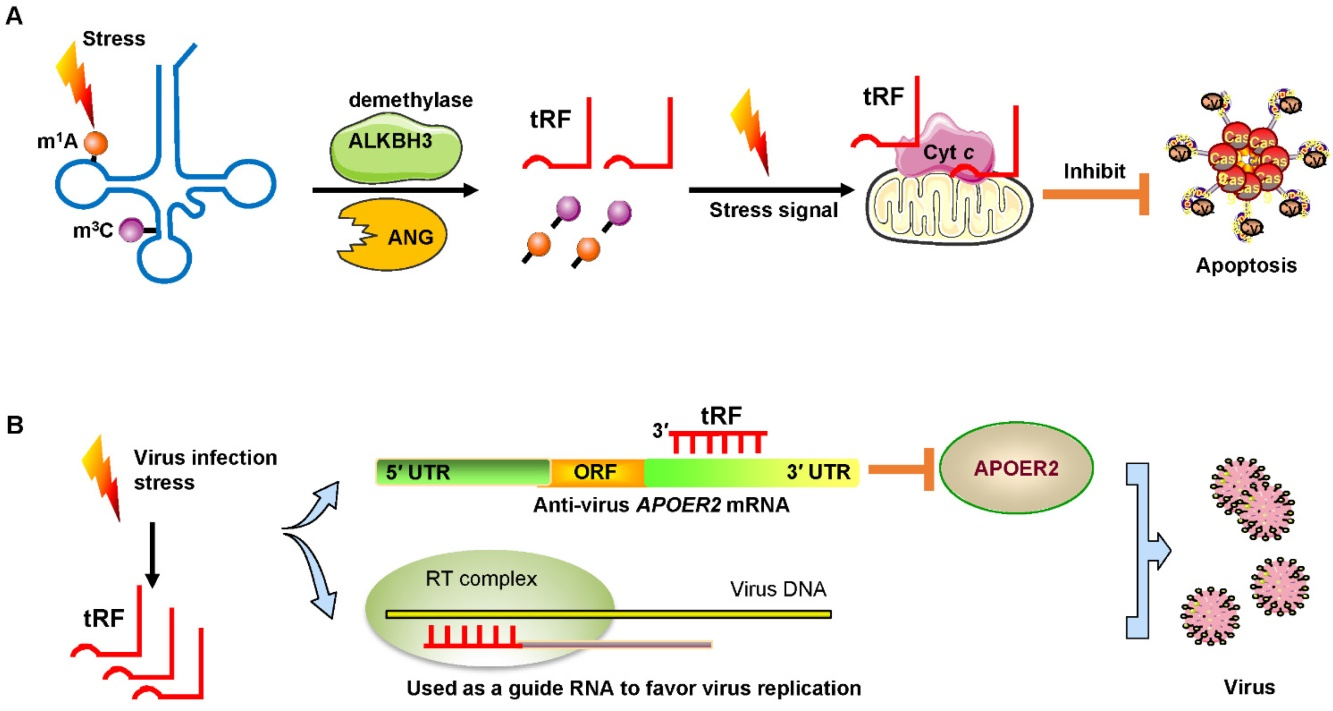
Signs of stress and viral infection. (A) Under stress, the demethylase ALKBH3 induces tRFs to interact with Cyt *c* to suppress cancer cell apoptosis. (B) Virus infection-induced stress leads to the induction of specific tRFs. Some tRFs recognize the target site in the 3′ UTR of anti-virus protein APOER2 mRNA. The suppression of APOER2 will promote virus replication. Other tRFs are used as guide RNAs to initiate viral reverse transcription. Abbreviations: ALKBH3, α-KG-dependent alkB homolog 3; ANG, Angiogenin; Cyt *c*, cytochrome *c*; UTR, untranslated region; ORF, Open reading frame; APOER2, apolipoprotein E receptor 2; RT, reverse transcription.

Under sodium arsenite stress, Chen et al. revealed that the demethylase α-KG-dependent alkB homolog 3 (ALKBH3) induced tRFs to interact with cytochrome *c* (Cyt *c*) to suppress cell apoptosis 55. ALKBH3 catalyzes the demethylation of 1-methyladenosine (m^1^A) and 3-methylcytidine (m^3^C) in tRNAs. Demethylated tRNAs are more sensitive to the cleavage of ANG and easily generate tRF-5^Gly-GCC^ 55, 56. These tRFs bound to Cyt *c* that was released from the mitochondria and then eventually strengthened ribosome assembly and finally prevented apoptosis of cervical cancer cells (**Figure 5A**).

Under oxidative stress, Gkatza et al. reported that the activity of the cytosine-5 RNA methyltransferase NSUN2 can be suppressed, leading to the reduction of tRNA methylation and then the intracellular biogenesis of tRFs related to the repression of protein synthesis 57.

### Promotion of virus replication

Recent studies have demonstrated that infecting host cells with respiratory syncytial virus (RSV) can initiate a stress response by mediating ANG digestion of tRNAs to generate increases in tRFs 58, 59. Viruses can exploit host tRFs as guide primers to improve their replication and promote the efficiency of infection 59, 60. Deng et al. convincingly demonstrated that a specific tRF originating from the 5′ end of tRNA^GluCTC^ (tRF-5^GluCTC^) was induced by RSV infection 61. The 3′-portion of tRF-5^GluCTC^ recognizes a target site in the 3′ UTR of apolipoprotein E receptor 2 (APOER2) mRNA (**Figure 5B**). APOER2 is a host anti-RSV protein whose inhibition favors RSV replication 61. As is well-known, the 5′-end of miRNAs is critical for their gene silencing effect 62. However, with regard to tRFs, the 3′-portion of tRF-5^GluCTC^ is important for gene targeting 63. Therefore, tRFs have different trans-silencing mechanisms than miRNAs. Zhou and his colleagues also found that RSV specifically led to the induction of two novel tRFs, tRF-5^GlyCCC^ and tRF-5^LysCTT^ 59. These tRFs play a significant role in promoting RSV replication and impact RSV-induced cytokines/chemokines. Ruggero et al. proved that tRF-3019 in host cells is thoroughly complementary to primer binding site (PBS) in retroviral RNA from human T-cell leukemia virus type 1 (HTLV-1) 60. Therefore, tRF-3019 could be utilized to guide RNA initiation of reverse transcription and increase virus amplification (**Figure 5B**).

Based on the above, we can see that tRFs play various roles in cancers and virus infection (**Table 1**).

## Clinical value of tRFs

In recent years, tRFs have become rising stars in the regulation of biological processes, and their deregulation has important clinical implications 64. Here, we describe the potential value of tRFs as diagnostic biomarkers and therapeutic targets (**Table 2**).

### Potential as diagnostic and prognostic biomarkers

As high-throughput sequencing technology has spread, an increasing number of studies have demonstrated that aberrant expression of tRFs contributes to carcinogenesis and could represent new biomarkers for diagnosis 65. tRFs carried by exosomes have been exploited as biomarkers and have been found to mediate communication between exosome-secreting cells and recipient cells 65. Zhu et al. found that patients with liver cancer exhibited significantly higher levels of tRF-5^GluCTC^ in plasma exosomes than healthy controls, indicating that exosomal tRFs in plasma can act as novel “liquid biopsy” biomarkers for the diagnosis of cancer 66. Sun et al. also showed that patients with high expression of tRF-27-ZDXPHO53KSN and tRF-30-JZOYJE22RR33 in serum obtain less benefit from trastuzumab-based therapy, indicating that these two tRFs could be explored as potential intervention targets and biomarkers in trastuzumab-resistant breast cancer 67. Biostatistical analysis revealed that i-tRF^GlyCCC^ levels were significantly lower in peripheral blood mononuclear cells (PBMCs) from chronic lymphocytic leukemia (CLL) patients and could be considered a screening biomarker 68. i-tRF^GlyGCC^ and i-tRF^PheGAA^ have been reported to have prognostic and diagnostic value in CLL, respectively 69, 70. In addition, Olvedy et al. demonstrated that patients prostate cancer in which the expression of tRF-315/tRF-544 was increased in tissues had obviously poorer progression-free survival (PFS) 71. These results indicated that abnormal tRFs could serve as fluid-based biomarkers for prospective screening research in the future.

### Potential clinical therapeutic targets

Aberrant expression of tRFs not only plays a diagnostic and prognostic role in cancers but also indicates their potential usage as therapy targets for the treatment of disease 72. For example, Kim et al. proved that tRF-3^LeuCAG^ resolved the hairpin structure of RPS28 mRNA and facilitated ribosome protein biogenesis to promote hepatocellular carcinoma growth 45. Using an antisense oligonucleotide to block tRF-3^LeuCAG^ prevents it from binding RPS28 mRNA, resulting in diminished ribosome biogenesis and apoptosis of hepatocellular carcinoma cells 45. This finding suggests the possibility that tRFs may be used as a therapeutic target in hepatocellular carcinoma therapy 45. Wang et al. reported that the combined utilization of an anti-tRF oligonucleotide and a small interfering RNA (siRNA) could downregulate RSV-induced tRF-5^GluCTC^ and eventually block RSV replication 63. Since the dysregulation of tRFs is closely related to cancers, cellular stress responses and virus infection in humans 73, tRFs may become new therapeutic targets for the treatment of diseases.

## Challenges and outlook

In this review, we mainly focused on the possible applications of tRFs in cancers and viral infections. However, tRFs also play roles in other types of diseases, including neurodegenerative and metabolic disorders 16, 57, 74, 75. The study of tRFs remains at an early stage; there are many problems that still need to be resolved.

First, how many additional classes of tRFs exist? For restriction sequencing technology, the current nomenclature of tRFs is based on the different cleavage loci of tRNAs or their origins in tRNAs that transfer a specific amino acid. The nomenclature and classification of tRFs are still rudimentary and cannot provide clear basic information about tRFs. Second, which mechanisms are crucial for tRF roles? The distribution of tRFs may contribute to their biological roles. A recent report suggested that tRF-5s are located mostly in the nucleus, whereas tRF-3s and tRF-1s are mostly cytoplasmic 15. The detailed mechanisms underlying the involvement of tRFs in cancers and stress may exceed our present knowledge and are worth more in-depth study in the future. Third, whether tRFs are safe and effective should be examined in the clinic. The applications of new bioinformatics techniques and additional experimental approaches, such as photoactivatable ribonucleoside-enhanced cross-linking and immunoprecipitation (PAR-CLIP) and cross-linking, ligation and sequencing of hybrids (CLASH), to understand the exact molecular mechanisms and the establishment of clinical indicators are critically important for the therapeutic application of tRFs.

Taken together, the evidence suggests that the roles and applications of tRFs still require intensive study. We are confident that future in-depth efforts will contribute to elucidating the mechanisms of tRFs and developing them as novel diagnostic biomarkers and therapeutic targets.

## Figures and Tables

**Table 1:** The mechanisms underlying the roles of tRFs in cancers and virus infections

Function	Biological effect	Mechanism	tRF name/ID	Cancer type/Virus infection	References
Regulation of mRNA stability	MiRNA-like actions	Conjunction with different Ago proteins or direct interaction with mRNAs	tRF^Leu^**-**3a/miR-1280,	Colorectal cancer,	23
			tRF**-**5^Gln^,	cervical carcinoma	25
			tRF-3^GlyGCC^,	B lymphoma	29
			tRF**-**3 (tRF^His-GTG^, tRF^Leu-CAG^),	Chronic lymphocytic leukemia	31
			tRF-3 (ts-3676, ts-4521*)*	Lung cancer	35
	Binds to RNA-binding proteins	Binding to YBX-1 or nucleolin	tRFs (tRF^Asp^, tRF^Glu^, tRF^Gly^, tRF^Tyr^)	Breast cancer	41, 44
Regulation of protein translation	Translational activation	Promotion of ribosome biogenesis	tRF-3^LeuCAG^	Hepatocellular carcinoma	45
			Gln19	Cervical carcinoma	47
	Translational inhibition	Displacement of translational initiation factors from mRNA or tRF-5 accumulation for the loss of m^5^C	mTOG-Ψ8	Myeloid malignancies	48
			tRF-5	Squamous tumor	51
Signs of cell stress and promotion of virus replication	Suppression of apoptosis	Interaction with Cyt c to suppress stress-induced cell apoptosis	tRF-5^GlyGCC^	Cervical carcinoma	55
	Promotion of virus replication	Targeting of anti-virus proteins or utilization as primers to favor virus replication	tRF-5^GlyCCC^, tRF-5^LysCTT^, tRF-5^GluCTC^,	Respiratory syncytial virus infection	59, 61
			tRF-3019	T-cell leukemia virus type 1 infection	60

**Table 2:** Clinical value of tRFs in cancers and virus infections

Function	Sample type	tRF name/ID	Cancer type/Virus infection	References
Potential diagnostic and prognostic predictive utility	Plasma (exosome)	tRF-5^GluCTC^	Liver cancer	66
	Serum	tRF-30-JZOYJE22RR33, tRF-27-ZDXPHO53KSN	Breast cancer	67
	Blood (peripheral blood mononuclear cells)	i-tRF-Gly^CCC^, i-tRF-Gly^GCC^, i-tRF-Phe^GAA^	Chronic lymphocytic leukemia	68-70
	Tissue	tRF-315, tRF-544	Prostate cancer	71
Potential clinical therapeutic targets	Cell, serum, patient-derived xenograft model	3′tRF-LeuCAG	Hepatocellular carcinoma	45
	Cell, virus	tRF-5^GluCTC^	Respiratory syncytial virus infection	63
